# Integrating omics databases for enhanced crop breeding

**DOI:** 10.1515/jib-2023-0012

**Published:** 2023-07-25

**Authors:** Haoyu Chao, Shilong Zhang, Yueming Hu, Qingyang Ni, Saige Xin, Liang Zhao, Vladimir A. Ivanisenko, Yuriy L. Orlov, Ming Chen

**Affiliations:** Department of Bioinformatics, College of Life Sciences, Zhejiang University, Hangzhou 310058, China; Institute of Cytology and Genetics, Siberian Branch of the Russian Academy of Sciences, Novosibirsk 630090, Russia; Agrarian and Technological Institute, Peoples’ Friendship University of Russia, Moscow 117198, Russia; The Digital Health Institute, I.M. Sechenov First Moscow State Medical University of the Russian Ministry of Health (Sechenov University), Moscow 119991, Russia

**Keywords:** crop plant breeding, data integration, databases, omics, plant biology

## Abstract

Crop plant breeding involves selecting and developing new plant varieties with desirable traits such as increased yield, improved disease resistance, and enhanced nutritional value. With the development of high-throughput technologies, such as genomics, transcriptomics, and metabolomics, crop breeding has entered a new era. However, to effectively use these technologies, integration of multi-omics data from different databases is required. Integration of omics data provides a comprehensive understanding of the biological processes underlying plant traits and their interactions. This review highlights the importance of integrating omics databases in crop plant breeding, discusses available omics data and databases, describes integration challenges, and highlights recent developments and potential benefits. Taken together, the integration of omics databases is a critical step towards enhancing crop plant breeding and improving global food security.

## 1 Introduction

**Figure 1:**
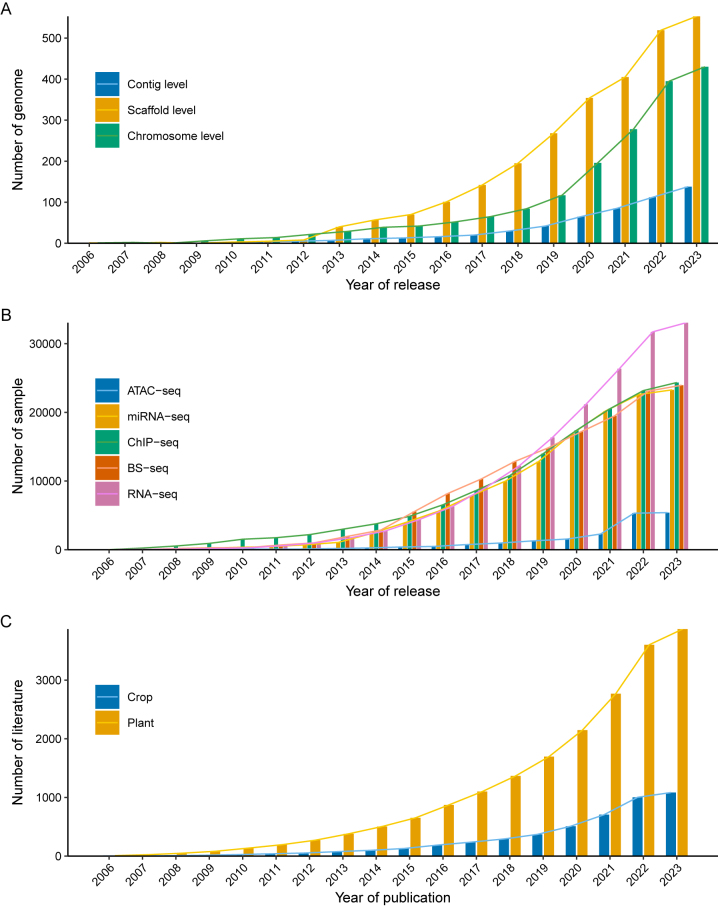
Statistics of land plant omics data and literature. (A) The number of completed genome assemblies for land plants. The data was downloaded from NCBI GENOME REPORTS, which filtered all genomes with the “land plants” tag and a genome size of less than 100 Mb. (B) The amount of epigenomic and transcriptomic data generated from land plants. The number of each omics sample was searched in the NCBI SRA database using a query such as “(((land plants [organism]) AND 2023)) AND RNA-seq [strategy]”. The sample size of “RNA-Seq” is the result after being reduced by 20 times. (C) The number of literatures on land plant omics research. The literature count was searched in the PubMed database by “omics plant” or “omics crop”.

Crop plant breeding is a complex and challenging process that requires the identification and selection of desirable traits such as increased yield [1], improved disease resistance [2], and enhanced nutritional value [3]. Over the years, traditional breeding methods have been used to develop new plant varieties by crossing plants with desirable traits to produce offspring with improved traits [4–6]. However, these methods are time-consuming and often limited by the genetic diversity of available plant species. In recent years, the emergence of high-throughput omics technologies has revolutionized crop plant breeding by providing vast amounts of data on the molecular mechanisms underlying plant development [7], and responses to environmental stresses [8]. Genomics is essential in crop breeding, allowing the identification of important genetic traits and accelerating the development of improved varieties. The number of sequenced crop genomes has continued to rapidly grow in recent years (Figure 1A), providing valuable resources for agricultural research. Additionally, epigenomics and transcriptomics have become increasingly important in crop breeding, providing insights into gene regulation and aiding in the identification of desirable traits [9, 10]. The SRA database has seen a continuous increase in epigenomic and transcriptomic data, further emphasizing the significance of these fields for crop breeding (Figure 1B). Proteomics and metabolomics have continued to develop in crop breeding, allowing for a deeper understanding of plant molecular mechanisms [11, 12].

These technologies have enabled the identification of key genes and pathways involved in crop traits, allowing breeders to select and develop new plant varieties with desirable traits more efficiently [4–6]. In recent years, there has been a significant increase in literature focused on the application of omics technologies in crop breeding (Figure 1C), highlighting the growing importance of these approaches in agricultural research. However, the effective use of omics technologies in crop plant breeding requires the integration of diverse datasets from different databases. Integration of omics data is crucial in providing a comprehensive understanding of the biological processes underlying plant traits and their interactions. In recent years, several omics databases have been developed to store and analyze large-scale omics data for different crop species, including rice (Table 1), maize [13], wheat [14], and soybean [15]. These databases provide a wealth of information on the genetic makeup, epigenome regulation, gene expression profiles, protein functions, and metabolic pathways of crops, which can be used to improve breeding programs.

**Table 1:** Statistics of rice omics database.

Database name	Omics type	Number of accessions	Released year	Link	Ref.
RAP-DB	Genome	1	2006	https://rapdb.dna.affrc.go.jp	[16]
BGI-RIS	Genome	1	2007	http://rice.genomics.org.cn	[17]
RGAP	Genome	1	2005	http://rice.uga.edu	[18]
RIGW	Genome	2	2020	http://rice.hzau.edu.cn/rice_rs3	[19]
RGI	Genome	16	2023	https://riceome.hzau.edu.cn	[20]
RPAN	Genome	3010	2016	https://cgm.sjtu.edu.cn/3kricedb	[21]
RiceENCODE	Epigenome	972	2021	http://glab.hzau.edu.cn/RiceENCODE	[22]
RiceXPro	Transcriptome	194	2011	https://ricexpro.dna.affrc.go.jp	[23]
RED	Transcriptome	284	2017	http://expression.ic4r.org	[24]
PPRD	Transcriptome	11,726	2022	https://plantrnadb.com/ricerna	[25]
RPMD	Proteome	38	2004	http://www.info.chi-biotech.cc	[26]
RKD	Proteome	1429	2007	https://ricephylogenomics.ucdavis.edu	[27]
MCDRP	Proteome	2400	2013	http://www.genomeindia.org/biocuration	[28]
RiceCyc	Metabolome	316	2013	http://pathway.gramene.org/gramene	[29]

The integration of omics databases can provide several benefits to crop plant breeding. Firstly, it can help to identify novel gene targets that are associated with desirable traits [30]. This can be achieved by integrating genomic, epigenomic, transcriptomic, proteomic, and metabolomic data to identify genes that are differentially expressed or are involved in key metabolic pathways. Secondly, it can help to develop predictive models for crop performance by integrating different omics data and environmental factors. These models can be used to predict the performance of new plant varieties under different environmental conditions and select the best performing varieties for further development [31]. Furthermore, the integration of omics databases can accelerate breeding cycles by providing breeders with a better understanding of the molecular mechanisms underlying crop traits [32]. This can help to reduce the time and cost required to develop new plant varieties with desirable traits. Finally, it can help to improve global food security by providing breeders with the tools and resources needed to develop new crop varieties that are more resilient to environmental stresses and can produce higher yields [33].

In this review, we aim to highlight the importance of integrating omics databases in crop plant breeding and discuss the current state of integration efforts. We will begin by discussing the different types of omics data available for crop plants, including genomic, epigenomic, transcriptomic, proteomic, and metabolomic data. We will then review the different databases that host these omics data and describe their features, strengths, and limitations. Next, we will discuss the challenges associated with integrating omics databases, such as data heterogeneity, scalability, and interoperability. Then, we will highlight some of the recent developments in omics data integration in crop plant breeding and the potential benefits of these efforts. Finally, we will discuss the use of machine learning algorithms and network analysis tools to integrate omics data and identify key genes and pathways associated with desirable traits. Overall, the integration of omics databases is a critical step towards enhancing crop plant breeding and improving global food security. The use of omics technologies and databases can provide breeders with the tools and resources needed to develop new crop varieties with desirable traits more efficiently and sustainably. The integration of omics databases is a rapidly evolving field, and future developments in this area are expected to further enhance our ability to develop crops that are more productive, resilient, and sustainable.

## 2 Omics data and databases for crop plants

**Figure 2:**
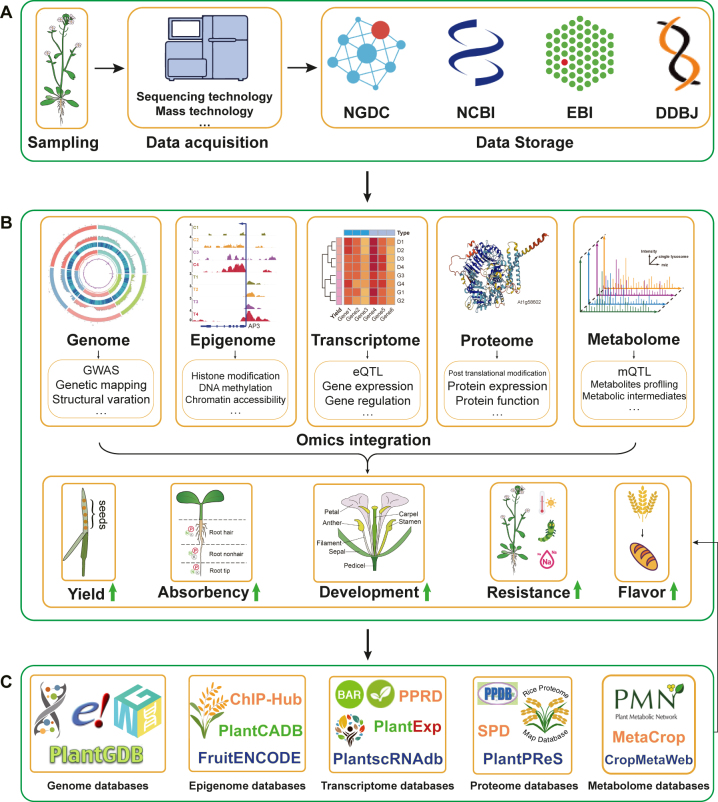
Generation, storage, mining, and integration of crop omics data. (A) Generation and storage of omics data in public databases. (B) Applications of five omics technologies (genomic, epigenomic, transcriptomic, proteomic, and metabolomic data) in crop breeding. (C) Construction of a secondary database based on mining of multi-omics data.

Crop plants are complex organisms that have undergone natural selection and human domestication. Omics technologies provide a powerful tool for investigating the genetic and molecular mechanisms underlying plant growth, development, and responses to environmental stresses [30, 31]. With the decreasing cost of high-throughput sequencing, an increasing amount of molecular information on crops is being obtained. This has led to the rapid establishment of large public databases for the sharing of bioinformatics data in various countries, such as the National Genomics Data Center (NGDC) [34], the National Center for Biotechnology Information (NCBI) [35], the DNA Data Bank of Japan (DDBJ) [36], and the European Bioinformatics Institute (EBI) [37] (Figure 2A).

Through omics data, crop shape can be improved, including increasing yield, enhancing the root system’s nutrient uptake ability, improving plant adaptability to the environment, resistance to adversity, and flavor, among other things (Figure 2B). With the expansion of biological big data, more and more secondary databases have been established to better integrate and analyze multi-omics data, thereby explaining the molecular mechanisms of crops at different levels (Figure 2C). In this article, we will first introduce the five main types of omics data commonly used in crop plant research: genomic, epigenomic, transcriptomic, proteomic, and metabolomic data.

Genomic information has proven to be an invaluable tool for crop improvement [38]. The identification of genes responsible for desirable traits such as resistance to diseases or high yield is facilitated by genomic data. The use of genomic databases such as NCBI Assembly [39], Genome Warehouse [40], EnsemblPlants [41], Phytozome [42], and PlantGDB [43] provides access to genome sequences, gene annotations, and functional annotations for many crop species, including rice, maize, soybean, wheat, and so on (Table 2). This information can help researchers develop molecular markers and breeding programs that produce improved crop varieties with enhanced characteristics. Furthermore, genomics can aid in understanding the evolution and domestication of crops, which can have implications for their conservation and management. Therefore, genomics is the cornerstone of omics research. Overall, genomics has the potential to transform agriculture by improving crop productivity, sustainability, and resilience, contributing to global food security.

**Table 2:** Statistics of comprehensive omics databases about plants.

Database name	Omics type	Number of accessions	Released year	Link	Ref.
Assembly	Genome	2662	2016	https://www.ncbi.nlm.nih.gov/assembly	[39]
GWH	Genome	1358	2021	https://ngdc.cncb.ac.cn/gwh	[40]
Phytozome	Genome	312	2012	https://phytozome-next.jgi.doe.gov	[42]
PlantGDB	Genome	187	2004	http://plantgdb.org	[43]
EnsemblPlants	Genome	134	2002	https://plants.ensembl.org/index.html	[41]
ChIP-Hub	Epigenome	>10,000	2022	https://biobigdata.nju.edu.cn/ChIPHub	[44]
PlantCADB	Epigenome	649	2022	https://bioinfor.nefu.edu.cn/PlantCADB	[45]
PlantExp	Transcriptome	131,423	2023	https://biotec.njau.edu.cn/plantExp	[46]
PPRD	Transcriptome	∼45,000	2022	http://ipf.sustech.edu.cn/pub/plantrna	[25]
Genevestigator	Transcriptome	>250,000	2006	https://genevestigator.com	[47]
ePlant	Transcriptome	>10,000	2005	http://bar.utoronto.ca	[48]
PlantGenIE	Transcriptome	35,533	2015	https://plantgenie.org	[49]
PsctH	Transcriptome	20	2021	http://jinlab.hzau.edu.cn/PsctH	[50]
PlantscRNAdb	Transcriptome	31	2021	http://ibi.zju.edu.cn/plantscrnadb	[51]
PCMDB	Transcriptome	22	2022	http://www.tobaccodb.org/pcmdb	[52]
PPDB	Proteome	>5000	2004	http://ppdb.tc.cornell.edu	[53]
PlantPReS	Proteome	>20,000	2016	http://www.proteome.ir/	[54]
PMN	Metabolome	9129	2021	https://plantcyc.org	[55]
MetaCrop	Metabolome	392	2008	https://metacrop.ipk-gatersleben.de	[56]

Epigenomic data can be integrated with other omics data to gain a more comprehensive understanding of the underlying biological processes. For example, integrating epigenomic data with transcriptomic data can provide insights into how changes in chromatin structure affect gene expression [57]. This can help identify key regulatory genes and pathways that can be targeted in crop breeding programs. Despite its potential, integrating epigenomic data poses unique challenges due to the complex nature of epigenetic modifications and the difficulty in accurately measuring them. However, recent advancements in high-throughput epigenomic technologies, such as ATAC-Seq [58], ChIP-seq [59] and BS-Seq [60], have made it possible to generate large amounts of epigenomic data in a cost-effective and efficient manner. Currently, there are Encyclopedia of DNA Elements (ENCODE) projects for human and mouse, but as yet there is no well-defined project for plants. In 2014, the international plant science community launched the Plant ENCODE project [61]. Since then, with the efforts of plant researchers worldwide, several ENCODE databases for various plant species have been established (Table 2), including RiceENCODE, which provides an important platform for studying the epigenome, genetic mechanisms, tissue specificity of rice. Additionally, FruitENCODE [62] has obtained various functional genomic data for 11 fleshy fruits, laying the groundwork for understanding the molecular regulation of fruit ripening. Moreover, comprehensive plant regulome databases called ChIP-Hub [44] and PlantCADB [45] also has been constructed (Table 2). In conclusion, incorporating epigenomic data into omics-based approaches can further enhance crop plant breeding by providing a more comprehensive understanding of the biological processes underlying desirable traits. By integrating diverse omics datasets, researchers can identify key regulatory genes and pathways that can be targeted to develop new plant varieties with improved yield, disease resistance, and nutritional value.

Transcriptomic data is crucial in advancing crop breeding by providing crucial insights into gene expression patterns in different tissues and under varying conditions. In addition to identifying differentially expressed genes, transcriptomic data can help researchers to understand the complex regulatory networks that control gene expression, including the involvement of non-coding RNAs (ncRNAs) such as long non-coding RNAs (lncRNAs) and microRNAs (miRNAs) [63]. These ncRNAs have emerged as important players in gene regulation and can significantly influence crop traits and responses to environmental stimuli. Besides, single-cell transcriptomic analysis is an emerging technology that allows researchers to study gene expression patterns at the level of individual cells [64]. This technology has revolutionized the field of transcriptomics, enabling researchers to identify rare cell types, map developmental trajectories, and uncover novel gene expression patterns that are masked in bulk transcriptomic analyses. By applying single-cell transcriptomics to crop plants, researchers can gain a more comprehensive understanding of gene expression patterns in different cell types and tissues, and the molecular mechanisms that govern crop growth and development. To access bulk transcriptomic data for crop plants, researchers can use established databases such as PlantExp [46], PPRD [25], Genevestigator [47], ePlant [48], and PlantGenIE [49], which provide a variety of transcriptomic data sets for different crop species (Table 2) Besides, with the widespread application of single-cell transcriptomic technology in plants, databases focused on plant single-cell transcriptomics, such as PsctH [50], PlantscRNAdb [51], and PCMDB [52] have been established successively (Table 2). These databases are critical resources that enable researchers to explore gene expression patterns across different tissues and under varying conditions. The availability of transcriptomic data sets from different crop species and tissues has greatly facilitated the identification of candidate genes and pathways for crop improvement, thereby enabling the development of more productive and resilient crop varieties.

Proteomic data is a valuable tool for understanding the protein content and function of crop plants. By using proteomic data, researchers can identify proteins involved in critical metabolic pathways or associated with specific traits. The Plant Proteome Database (PPDB) [53], Plant stress proteome database (PlantPReS) [54], Rice Proteome Database (RPD) [65], Soybean Proteome Database (SPD) [66], are among the most commonly used proteomic databases for crop plants (Table 2), providing access to proteomic datasets for various crop species, including protein sequences, structures, and functional annotations. Proteomics plays a critical role in crop science research, as it provides researchers with a comprehensive view of the protein content of crop plants. This information can be used to improve crop yield and quality, increase stress tolerance, and develop new crop varieties with improved traits. For example, proteomic data has been used to identify proteins associated with abiotic stress responses, such as drought or salinity [67], and to identify proteins involved in plant-microbe interactions [68], such as those associated with disease resistance. In addition, proteomic data can be used to identify proteins associated with specific crop traits, such as those related to nutritional value or flavor. This information can be used to develop crops with enhanced nutritional content or improved flavor profiles [69], which can increase their value to consumers. Looking forward, proteomics will continue to play an important role in crop science research, as new technologies and methods are developed to analyze and interpret proteomic data. These advances will allow researchers to gain a more detailed understanding of the protein content and function of crop plants, which can be used to develop new crop varieties that are more resilient, productive, and sustainable.

Metabolomics is a powerful tool for investigating the genetic basis of metabolic variation, providing insights into the complex biochemical cascades that connect the genome, transcriptome, and proteome to phenotype [70]. By analyzing a wide range of sample types, including primary cells, tissues, biofluids, and entire organisms, metabolomics can determine the relative and absolute amounts of various metabolites, such as sugars, lipids, amino acids, and nucleotides. In crop science research, metabolomics is also an important tool that offers a comprehensive view of the metabolite content and function of crop plants. Using metabolomics data, researchers can identify metabolites that are involved in critical metabolic pathways or associated with specific traits in crops [71]. Several commonly used metabolomic databases for crop plants include Plant Metabolic Network (PMN) [55], and MetaCrop [56], a detailed database of crop plant metabolism (Table 2). Metabolomics not only aids in identifying individual metabolites but also contributes to the development of crops that have superior stress tolerance or nutritional content. One application of metabolomics is the detection of biomarkers linked to abiotic stress responses, which can then be utilized to cultivate crops that are more resistant to these conditions. Additionally, metabolomics can determine metabolic pathways that influence specific crop traits, such as those affecting nutritional value or flavor, and can be utilized to create crops with superior nutritional content or flavor profiles.

Different from the micro-level molecular omics, macro-level crop phenomics is the focal point of breeders. Therefore, in recent years, phenomics has also emerged as a field of study. Phenomics refers to a comprehensive and systematic approach to studying and describing the phenotypes of organisms, combining the terms “phenotype” and “omics”. Phenomics methods often utilize high-throughput techniques and large-scale data analysis to collect and analyze phenotype data. These techniques may include image analysis, genomics, transcriptomics, metabolomics, proteomics, and others, in order to obtain comprehensive information about an individual’s phenotype. By integrating phenotype data with genomic and environmental data, researchers can identify genes or environmental factors associated with specific phenotypic features, revealing the genetic basis and regulatory mechanisms underlying phenotypes.

In conclusion, the application of omics technologies and databases offers a powerful tool for investigating the intricate genetic and molecular mechanisms that underlie the growth, development, and responses of plants to environmental stresses. The diverse types of omics data complement each other in providing a comprehensive view of the molecular processes involved in crop plants. However, it is important to consider the distinct features, strengths, and limitations of the different databases that host these omics data when selecting an appropriate database for a particular research question. A judicious selection of a database can facilitate the integration of omics data, and provide valuable insights into the biology of crop plants, leading to the development of new crop varieties with desirable traits at an accelerated pace.

## 3 Challenges of integrating omics from databases

**Figure 3:**
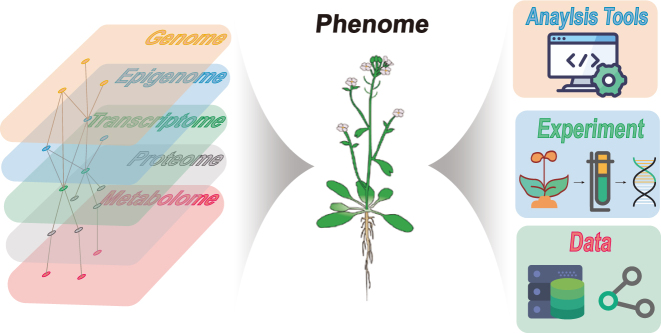
Challenges of integrating omics data. The left side represents the challenges of integrating omics data stored in various databases, where the dots and lines indicate the potential mutual regulation of different omics levels. The right side represents the challenges of data processing at different levels, including challenges in using bioinformatics analysis tools for data processing, challenges in obtaining experimental data, and challenges in data storage and sharing. The middle section represents the growth phenotype of plants.

Integrating omics data from different databases presents several challenges due to the heterogeneity of the data, differences in data formats, and varying levels of data quality. One of the primary challenges is the integration of data from different omics technologies, which often use different data formats and produce data with different levels of complexity (Figure 3). For example, genomic data typically consists of large, complex data sets, while proteomic data may contain information on thousands of individual proteins. Overcoming these challenges requires the development of standardized data formats and integration tools that can handle diverse data types.

Another challenge associated with integrating omics databases is scalability. As the number of omics data generated increases, it becomes increasingly difficult to store, process, and analyze the data. For example, a single genome sequence for a crop plant may require hundreds of gigabytes of storage, while a large-scale proteomic study may generate terabytes of data. Scalability can be addressed through the use of cloud-based storage and computing resources, as well as the development of efficient algorithms and data compression techniques.

Interoperability is another challenge that arises when integrating omics databases. Different databases may use different ontologies and vocabularies to describe the same biological concepts, making it difficult to integrate data from different sources. Furthermore, data may be stored in different formats or with different levels of annotation, making it difficult to compare and analyze the data. This is especially common in single-cell transcriptome studies. Interoperability can be improved through the use of common data standards and the development of ontology-based integration tools that can map data from different sources onto a common framework.

In conclusion, integrating omics data from different databases presents several challenges that must be addressed in order to fully exploit the potential of these data for crop plant research. Overcoming these challenges requires the development of standardized data formats, scalable storage and computing resources, and ontology-based integration tools. With the development of these tools and resources, the integration of omics databases can provide valuable insights into the biology of crop plants and help to accelerate the development of new crop varieties with desirable traits.

## 4 Recent developments in omics data integration for crop plant breeding

Recent developments in omics data integration for crop plant breeding have shown promise in accelerating the development of new crop varieties with desirable traits [72, 73]. One approach is the use of machine learning algorithms to integrate data from different omics technologies and predict the performance of different crop varieties under different environmental conditions. Today, in the field of computer science, machine learning has produced numerous excellent algorithms and frameworks. Currently, machine learning methods applied in biological research can be roughly categorized into unsupervised, supervised, and reinforcement learning.

Unsupervised learning aims to extract latent data features or structures from unlabeled biological data. For example, unsupervised dimensionality reduction techniques such as Principal Component Analysis (PCA) and Singular Value Decomposition (SVD) can be applied to crop sequencing and quantitative samples, along with clustering methods like K-means clustering and hierarchical clustering. These approaches can help us better understand the characteristics of the omics data. In contrast, supervised learning requires the use of labeled training data, where the input and output are known, to build models. These models are then used to predict and classify new inputs, using algorithms such as K-Nearest Neighbors (KNN), Support Vector Machines (SVM), Random Forests, Decision Trees, Naive Bayes, etc. Supervised learning can be applied, for example, to differentiate between good and bad genotypes based on molecular data. Finally, unlike unsupervised and supervised learning, reinforcement learning focuses more on iterative experimentation (trial and error) and delayed rewards. It continuously optimizes the correspondence between states and actions based on feedback (rewards) provided by the environment. One application of reinforcement learning is in protein structure prediction [74].

A recent study used machine learning algorithms to integrate genomic and phenotypic data and predict the performance of different varieties under drought conditions [75, 76]. The results showed that the algorithm was able to accurately predict the performance of different varieties, and identified several new candidate genes that may be involved in drought tolerance.

Another approach is the use of multi-omics data integration to identify key regulatory networks and pathways that underlie specific traits or responses to environmental stresses [77]. For example, a recent study used multi-omics data integration to identify key regulatory networks involved in salt stress tolerance in tomato [78]. The study integrated transcriptomic, proteomic, and metabolomic data and identified several key regulatory pathways involved in salt stress tolerance, including the production of osmoprotectants and the regulation of ion transport. Overall, The potential benefits of omics data integration for crop plant breeding are numerous [79]. By integrating data from different omics technologies, researchers can gain a more comprehensive understanding of the molecular processes underlying crop growth, development, and responses to environmental stresses [80]. The functional annotation of the plant gene and gene-phenotype association could be found by new text mining tools [81, 82]. This knowledge can be used to develop new crop varieties with improved yields, disease resistance, and stress tolerance, as well as to identify new targets for crop improvement. In addition, the integration of omics data can help to accelerate the breeding process by reducing the time and resources required for traditional breeding methods.

## 5 Discussion

The integration of omics data in crop plant breeding has brought about tremendous advances in the development of new crop varieties that possess desirable traits such as increased yield, improved disease resistance, and enhanced nutritional value [1–3]. The integration of diverse omics datasets from different databases provides a comprehensive understanding of the underlying biological processes and interactions that influence plant traits. Machine learning algorithms and multi-omics data integration have emerged as powerful tools for analyzing and interpreting omics data, enabling the development of predictive models that can accelerate the breeding process and reduce the time and resources required for traditional breeding methods. These models can identify genetic factors underlying desirable traits and predict the performance of different varieties under specific environmental conditions, allowing for more efficient and effective selection and crossing of plants.

Moreover, the integration of omics data can be used to develop new crop varieties with improved yields, disease resistance, and stress tolerance, as well as to identify new targets for crop improvement. However, the integration of omics data poses several challenges, including the sheer volume of data generated by different omics technologies, the complexity of integrating multiple omics data sets, and the need for advanced computational tools and expertise to analyze and interpret these data. Additionally, the ethical and social considerations associated with the use of omics data in crop breeding cannot be ignored. The development of new crop varieties with desirable traits can have significant impacts on the environment, local communities, and the wider agricultural system [83]. Therefore, transparent and inclusive decision-making processes that involve all stakeholders, including farmers, consumers, and policymakers, are essential.

In conclusion, the integration of omics data in crop plant breeding is a critical step towards enhancing crop productivity and improving global food security. Addressing the challenges associated with the integration of omics data will require collaboration and coordination among researchers, policymakers, and stakeholders across the agricultural sector. The benefits of omics data integration in crop breeding are enormous, and it is essential that efforts are made to leverage these technologies for the betterment of humanity.
